# VARIATIONS ACROSS 11 DEPARTMENT OF VETERANS AFFAIRS HOSPITAL-IN-HOME PROGRAMS IN 2021–2022

**DOI:** 10.1093/geroni/igad104.0539

**Published:** 2023-12-21

**Authors:** Orna Intrator, Shubing Cai, Jiejin Li, Rajesh Makineni, William Hung

**Affiliations:** Department of Veterans Affairs, Rochester, New York, United States; University of Rochester Medical Center, Rochester, New York, United States; Canandaigua VA Medical center, Canandaigua, New York, United States; University of Rochester, Rochester, New York, United States; Icahn School of Medicine at Mount Sinai, New York City, New York, United States

## Abstract

The Department of Veterans Affairs’ (VA) Hospital-In-Home (HIH) program provides Veterans with the option to receive acute level services in the comforts of their own homes. A total of 11 HIH programs operated between fiscal years 2021 and 2022. We examined the volume of the programs and average risk of one-year mortality (using the Care Assessment of Needs measure) among each program’s participants and characteristics of HIH stays (length-of-stay [LOS] and discharge destination) across program sites and program types (complementary- admissions from hospital and substitutive- other admissions) using VA administrative data linked with Medicare claims. The number of patients served in a year varied between 36 and 680 with 6 programs having <200 patients. Substitutive HIH was the dominant HIH type in 8 programs (>50% of patients) while only one program had clearly more complementary HIH (87%) and only 9 programs had complementary HIH. Average length-of-stay (los) in HIH was 20.5 days (SD=24.4 days, median=13 days), with averages across programs ranging between 3 - 36 days, 6 programs with average los<13 days and 5 programs with average los >19 days. Predicted risk of death within one-year varied: among substitutive HIH, 7 programs’ average risk was > 20% and 4 programs’ average risk was <15%. Among complementary HIH only one program’s risk was <15% and the rest was >20%. Substitutive HIH programs discharged 2-10% of Veterans to hospital, while complementary programs discharged 11-23% to hospital. Interviews with program staff will help elucidate reasons for differences and inform expected outcomes.

